# Supplementary material for: A protocol for a controlled, pre-post intervention study to change attitudes toward child marriage in Southwestern Nigeria using targeted radio programming
Source: PLoS One. 2023 May 15;18(5):e0285902. doi: 10.1371/journal.pone.0285902 (PMC10184895; doi:10.1371/journal.pone.0285902)
Supplement: Attachment — Submitted filename: GCC-response-to-reviewers-17March.docx [file pone.0285902.s001.docx]

To the Editor,

Thank you for facilitating the peer-review process at a time when finding reviewers is becoming increasingly challenging. Our point-by-point responses to both reviewers’ comments on our study protocol follow. We appreciate the opportunity to resubmit this work.

Reviewer 1

1. It would be helpful to include information on the listenership of the radio stations in Ibadan and then verify access to that radio station in Akure to ensure that there is no contamination or information leak baring in mind the era of internet radio.

Thank you for this suggestion. We verified that the broadcast range of the stations we plan to use does not extend to Akure but are aware that online access may lead to some spillover of the intervention. We acknowledge this possibility, and why we believe it is a small possibility, on page 6.

Reviewer 2

1. The last sentence under financial disclosure is somewhat confusing. Rephrase.

The financial disclosure statement was part of the webform, not the actual study protocol, and has been rephrased in the revised version.

2. Provide references for the last two sentences in the second paragraph [of the introduction].

We added two citations to support the points made in the sentences indicated:

First, a study conducted among refugees in Lebanon that documents child marriage as a response to the perceived risks of sexual violence: Mourtada R et al. A qualitative study exploring child marriage practices among Syrian conflict-affected populations in Lebanon. *Conflict and Health*. 2017;11(27).

Second, a study that highlights child marriage as a solution to the risk of premarital sexual activity in Bali: Horii H. Child Marriage as a ‘Solution’ to Modern Youth in Bali. *Progress in Development Studies*. 2020;20(4):282-295.

3. Objectives should come at the end of the introduction and not under methods. Also, the design of the radio programmes is also an objective and should be listed as such.

The organization of the manuscript aligns with PLOS ONE’s standards for protocols and, in our opinion, facilitates a higher degree of readability. (If this were a manuscript describing the results of the study rather than a protocol, we would be more inclined to agree with the reviewer that the study’s objectives should be stated earlier in the paper.) Moreover, we feel that it would add confusion to the paper if we were to state objectives regarding a radio intervention before we have actually described that intervention. For these reasons, we have not changed the organization.

4. Both objectives stated currently are ‘double barreled’. For example, objective 1 seek to determine the effective reach of the radio programmes (an objective on its own) and also determine improvement in knowledge (another objective). I advise that these should be separated.

We rephrased the objectives as recommended.

5. The study settings should be the first subsection here [under the Methods heading]. The intervention should come after inclusion criteria.

We moved the subsection on study setting as recommended.

6. The authors repeatedly used the term ‘random selection’ which should be correctly termed ‘simple random sampling’. Simple random sampling is a known sampling method, but random selection is not. This should be corrected appropriately.

We respectfully disagree with the reviewer. We will use a multi-stage cluster sampling approach, which is distinct from simple random sampling. Simple random sampling means that each person in the target population has an equal probability of being selected to participate in the study. In our case, the target population is Hausa persons living in Ibadan and Akure. However, as a result of our study design, not every Hausa person residing in those two cities has an equal probability of being selected. For example, we are only sampling from within specific neighborhoods with a high density of Hausa residents. This means that Hausa persons living elsewhere in these cities will not be selected, thus the probabilities of selection are not equal for every person in the target population and our sampling plan does not reflect “simple random sampling”. Moreover, our sampling plan will randomly select households within neighborhoods in both cities, meaning that Hausa persons who do not reside in selected households do not have an equal probability of being selected to participate.

We will use a multi-stage cluster sampling approach as described in the protocol. In principle, the sampling plan we will use is very similar to that used by Demographic and Health Surveys, which also do not use or describe their approach as simple random sampling. We have added the phrase “cluster sampling” to make this clearer.

For further information, please see: <https://www.qualtrics.com/au/experience-management/research/simple-random-sampling/>

7. Individuals of Hausa ethnicity should be added as a criteria (since they are the target) and not just those living in Hausa community, since other ethnic groups, especially the Yoruba hosts also live there.

We agree with the reviewer. The baseline survey questionnaire asks people to report their ethnicity, and identifying as Hausa is a criterion for inclusion in this study. The protocol has been updated to make this clearer. Please see page 8 for the updated text.

8. The second and third statements under this section [inclusion criteria] appear contradictory, kindly revisit.

We agree that the phrasing was confusing. We have omitted these statements entirely.

9. Is there any plan to include Hausa religious and traditional leaders in this study due to their influence in promoting societal norms that affect parents’ choices about the girl child marriage?

As noted in the protocol, our study is being guided by a Local Advisory Committee composed of ten Hausa community leaders from Ibadan, including religious authorities. These individuals will not be deliberately included in the survey, though there is a possibility that they could be selected based on the sampling procedures described. Rather, the Local Advisory Committee is contributing to the development of the intervention to ensure that the content is culturally appropriate and compelling.

A related qualitative study has already been undertaken in Ibadan and included key informants from the Hausa community, including religious leaders. Thus, prominent religious figures and other leaders have weighed in on these issues and their perspectives will inform the development of survey questions and the development of the radio intervention.

10. A mix of British and American English were used in the write up. Choose one of the English versions.

The first three authors are based in Nigeria and the corresponding author is based in Canada, hence the subtle differences in English language usage. We have updated the manuscript to use British English throughout.
